# Diverse expression of selected cytokines and proteinases in synovial fluid obtained from osteoarthritic and healthy human knee joints

**DOI:** 10.1186/s40001-014-0065-5

**Published:** 2014-11-29

**Authors:** Martin Sauerschnig, Josef Stolberg-Stolberg, Anne Schulze, Gian Max Salzmann, Carsten Perka, Christian Jiri Dynybil

**Affiliations:** Department of Trauma Surgery, Klinikum rechts der Isar, Technische Universitaet Muenchen, Munich, Germany; Centrum für Muskuloskeletale Chirurgie, Charité-Universitätsmedizin Berlin, Berlin, Germany; Department of Orthopaedic and Trauma Surgery, Albert-Ludwigs University Freiburg, Freiburg im Breisgau, Germany

**Keywords:** Cytokines, Proteinases, Osteoarthritis, Cartilage, Synovial fluid, Biomarkers

## Abstract

**Background:**

Osteoarthritis (OA) is defined by signs and symptoms of inflammation within the affected joint. The aim of this study is to determine the mRNA expression levels of selected cytokines and matrix-metalloproteinases of cells found in synovial fluid (SF) obtained from osteoarthritic knee joints compared to healthy controls.

**Methods:**

SF was obtained from 40 patients undergoing total knee arthroplasty due to evident OA and from 10 healthy controls. Expression of TNF-α, IL-1β, MMP-1 and MMP-3 was assayed among both groups by performing qPCR. Patients were configured concerning age, gender and BMI.

**Results:**

IL-1β, MMP-1 and MMP-3 showed significantly higher expression among the OA group compared to control (*P* < 0.001). Strong correlation appeared between expression of MMP-1 and MMP-3 among OA patients (r = 0.856); no correlation was found between age, gender or BMI and cytokine/proteinase expression. Expression of IL-1β, MMP-1 and MMP-3 within SF was elevated in OA-patients.

**Conclusion:**

Consequently, cells within SF expressing cytokines and proteinases may play a relevant role in the progression of joint destruction. Considering the fact that SF in an OA joint comprises abnormal amounts of detrimental bioactive proteins, temporary clearance, dilution or suppression/modulation by means of lavage or disease-modifying medication may be promising to constitute interim relief or even postpone disease progression due to decreased inflammatory and/or degrading activity within the articular environment.

## Background

Osteoarthritis (OA) is characterized by slowly progressing destruction of the articular cartilage as well as an inflammatory reaction within the realms of the affected joint [1]. OA represents the most common joint disorder in the world and results in pain, deformity and gradual loss of joint function [1-3]. The origin includes mechanical injury, genetic factors and aging [4]. Disease progression is characterized by formation of osteophytes, subchondral bone cysts, increased thickness of the subchondral bone plate and loss of articular cartilage [5]. The exact etiology of OA, however, is not fully understood.

Synovial inflammation is commonly found in early stage OA and is characterized by joint swelling, redness, heat and pain [6,7]. The synovium is infiltrated by an increased number of synovial lining cells and immune cells such as macrophages, mast cells, T- and B-lymphocytes and plasma cells. Activated synovial cells, immune cells and chondrocytes produce large quantities of matrix metalloproteinases (MMPs) -1, -3, -9, -13 and ADAMTS, a disintegrin and metalloproteinase with thrombospondin motifs [8]. These proteolytic enzymes eventually degrade extracellular matrix macromolecules and cause a loss of cartilage integrity and biomechanical strength [9-11].

Proinflammatory cytokines act as soluble mediators and favor catabolic activities in articular cartilage [12,13]. Cytokine production is associated with certain immune cells while their specific origin in OA, however, is still unclear [14]. The proinflammatory cytokines IL-1β and TNF-α are found in elevated concentration in synovial fluid (SF) and joint tissue of OA patients. Both activate the JNK, p38 MAPK and NF-κB signaling pathways in chondrocytes [15,16]. They are capable of suppressing proteoglycan, collagen II and aggrecan expression while stimulating MMP expression and the production of further catabolic cytokines such as IL-6, IL-8, MCP-1 and CCL-5 [17].

OA is no longer understood as a disease merely caused by wear and tear but rather as an inflammatory process [18]. Definition of baseline cytokine and proteinase levels and their mRNA concentration in SF of healthy and OA patients is crucial to evaluate the potential for diagnosing early OA using cytokines as biomarkers [19]. The objective of this study was to elucidate whether the cells in the SF are producing the detrimental proteins mentioned above. Thus, we aimed to quantify mRNA expression patterns of the main inflammatory mediators IL-1β and TNF-α and degradative enzymes MMP-1 and MMP-3 within synovial fluid of OA patients compared to healthy controls. Such information would further define the role of the mentioned cytokines and proteases within the complex cascades of incidental/progressive OA and could strengthen their potential as biomarkers and/or novel therapeutic targets.

## Methods

### Patients and donors

Patient and donor characteristics are presented in Table 1. A total of 40 patients (18 females, 22 males) with clinically as well as radiographically-evident OA were included and SF was obtained prior to total knee arthroplasty (TKA). Furthermore, SF was obtained from a total of 10 controls/donors (4 females, 6 males) with no history of joint surgery or any cartilage/OA-related disease, within 4 hours after death due to insult or atraumatic intracranial bleeding. SF harvesting among patients as well as controls was performed utilizing a sterile syringe with the needle injected into the knee joint using a standardized anterolateral portal, strictly avoiding hemarthrosis. Single incision arthrotomy allowed then for direct visualization of the knee joint and cartilage evaluation was performed by an experienced orthopedic surgeon in order to exclude the presence of OA.

**Table 1:** **Osteoarthritis (OA) patient and control characteristics**

	**OA patients**	**Control (donors)**	**Total**
Gender			
Male	22	6	28
Female	18	4	22
Total	40	10	50
Age			
Mean	67.88 ± 10.56	51.4 ± 6.36	64.58 ± 11.86
Range	39 to 99	42 to 62	39 to 99
Body Mass Index			
Mean	29.28 ± 5.77	25.26 ± 1.93	28.48 ± 5.47
Range	21.1 to 44.2	22.2 to 28.3	21.1 to 44.2

Gender (male/female), age and body mass index (BMI) at the time of examination among OA patients and controls, mean ± SD. Significance provided within results section.

The study was performed in accordance with the Declaration of Helsinki. Informed consent from each patient as well as approval from the local ethics committee was obtained prior to investigation (Ethics commission, Charité, Berlin, EA1/083/09).

### RNA extraction

After centrifugation of SF from OA patients and controls directly after harvest, the cell-pellet was disrupted in RA-1 lysis buffer (Macherey-Nagel, Düren, Germany) and stored at −80°C until further analysis. Total RNA was then isolated from the cell-pellet yielded using the NucleoSpin RNA II® kit (Macherey-Nagel, Düren, Germany) according to the manufacturer’s protocol.

### Reverse transcription

A volume of 8 μl mRNA template, 1 μl 10 mM Deoxynucleotide-Mix (dNTP-Mix, Invitrogen Life Technologies, Paisley, UK) and 1 μl random primer (Invitrogen Life Technologies, Paisley, UK) was incubated for 5 minutes at 65°C in a thermocycler (Eppendorf, Hamburg, Germany) and then cooled down to 4°C. A total of 9.5 μl reaction mix consisting of 4 μl reaction buffer (M-MLV RT 5x Reaktionspuffer, Promega GmbH, Mannheim, Deutschland), 1 μl RNase inhibitor (RNase Inhibitor 40 U/μl, Promega GmbH, Mannheim, Deutschland) and 4.5 μl H_2_O were added and incubated at 42°C for 2 minutes. After adding 1 μl of reverse transcriptase (M-MLV RT(H-) 200 U/μl, Promega GmbH, Mannheim, Deutschland), an incubation period of 50 minutes at 42°C was followed by 15 minutes at 70°C, producing the cDNA to be further analyzed via qPCR. After cooling to 4°C, residual RNA was eliminated using 1 μl of RNase (RNse H 250 U/μl, Promega GmbH, Mannheim, Deutschland) for 20 minutes at 37°C.

### Quantitative polymerase chain reaction (qRT-PCR)

Expression profiles were assessed via qRT-PCR on an iCycler detection system (Bio-Rad, Munich, Germany) using SYBR Green (Applied Biosystems, Foster City, CA, USA) as a fluorescent reporter. Primers were designed by TIB MOLBIOL (Syntheselabor GmbH, Berlin, Germany) and tested with the Basic Local Alignment Search Tool (BLAST). The human primer sequences used were as follows:

IL-1β: 5′-CAGGGACAGGATATGGAGCAA-3′ (forward)

5′-GCAGACTCAAATTCCAGCTTGTTA-3′ (reverse)

TNF-α: 5′-CTTCTCCTTCCTGATCGTGGC-3′ (forward)

5′-GGGTTTGCTACAACATGGGC-3′ (reverse)

MMP-1: 5′-CCGGTTTTTCAAAGGGAATAAGTA-3′ (forward)

5′-CGATATGCTTCACAGTTCTAGGGAA-3′ (reverse)

MMP-3: 5′-AAACCCACCTTACATACAGGATTG-3′ (forward)

5′-AAGTCTCCATGTTCTCTAACTGCA-3′ (reverse)

GAPDH: 5′-CATGTTCGTCATGGGTGTG-3′ (forward)

5′-GGCAGTGATGGCATGGACTG-3′ (reverse)

The 25 μl reaction-mix consisted of 1.0 μl cDNA template, 12.5 μl iQ Supermix (Bio-Rad, Munich, Germany), 0.5 μl primer forward (10 μM), 0.5 μl primer reverse (10 μM), 1.0 μl SYBR-Green (Applied Biosystems, Foster City, CA, USA) and 9.5 μl DNase-free water. iCycler settings involved activation of the polymerase reaction at 95°C (15 minutes), followed by 38 cycles of denaturation at 94°C (30 seconds), primer annealing at 59°C according to primer-specific requirements (40 seconds), elongation at 72°C (30 seconds) and fluorescence detection at 80°C (15 seconds). GAPDH was used as an internal control and all reactions were performed in triplicates followed by a specific melting curve analysis of each assay at 60°C to 95°C (30 seconds). Quantification of mRNA expression was assessed by the 2- ΔΔCT method [20].

### Statistics

The major determinant for outcome comparison was the expression level of the targeted cytokines and proteases according to the groups described above. Statistical analysis was performed using the software package SPSS version 17 (SPSS Inc., Chicago, IL, USA). All data were tested for normal distribution using the Kolmogorov-Smirnov test. The Mann-Whitney *U*-test was performed to assess differences in expression levels between groups corrected for cofounding variables (age, gender, BMI). Furthermore, Fisher’s exact test was conducted to compare the frequency of detectable expressions between the groups. To control the family-wise error rate, the Bonferroni correction was applied. Unless stated otherwise, descriptive results were demonstrated as the mean ± standard deviation (SD). Significance was set at *P* < 0.05 for all tests.

## Results

### Participant characteristics

An overview of participant characteristics is given in Table 1. All patients showed clinically and radiographically-evident knee OA. Controls showed no history of knee-related pathologies and there was no intraarticular, no ligamentous and particularly no meniscal or cartilage pathology to be found in any control knee joint and the surrounding soft tissues were also without pathological findings. There was a significant overall group difference for age and BMI (*P* < 0.05) between groups.

### qPCR outcome

IL-1β, MMP-1 and MMP-3 showed significantly higher expression levels among the OA group compared to the control (*P* < 0.001) while TNF-α expression differences never reached the level of significance (*P* = 0.077). Median and interquartile ranges (IQR: 25th to 75th percentile) are reported to depict differences in distribution of MMP-1, MMP-3, IL-1β and TNF-α between patients and donors/controls and an overview is given in Table 2.

**Table 2:** **Expression levels in osteoarthritis (OA) patients and controls**

		**Median**	**Interquartile range**		***P*** **-value**
			**25**	**75**	
MMP-1	OA SF	1.48	0.71	3.77	< 0.001
	Donor SF	< 0.01	< 0.01	< 0.01	
MMP-3	OA SF	3.78	1.49	8.64	< 0.001
	Donor SF	< 0.0	< 0.01	< 0.01	
IL-1β	OA SF	1.41	0.72	4.09	< 0.001
	Donor SF	< 0.01	< 0.01	1.91	
TNF-α	OA SF	2.57	1.22	5.59	0.077
	Donor SF	< 0.01	< 0.01	6.27	

Detectable expression levels of MMP-1, MMP-3, IL-1β and TNF-α within synovial fluid (SF) yield from affected patients (OA) and controls. Median and interquartile ranges (IQR: 25th to 75th percentile) are reported to depict differences in distribution.

**Figure 1:**
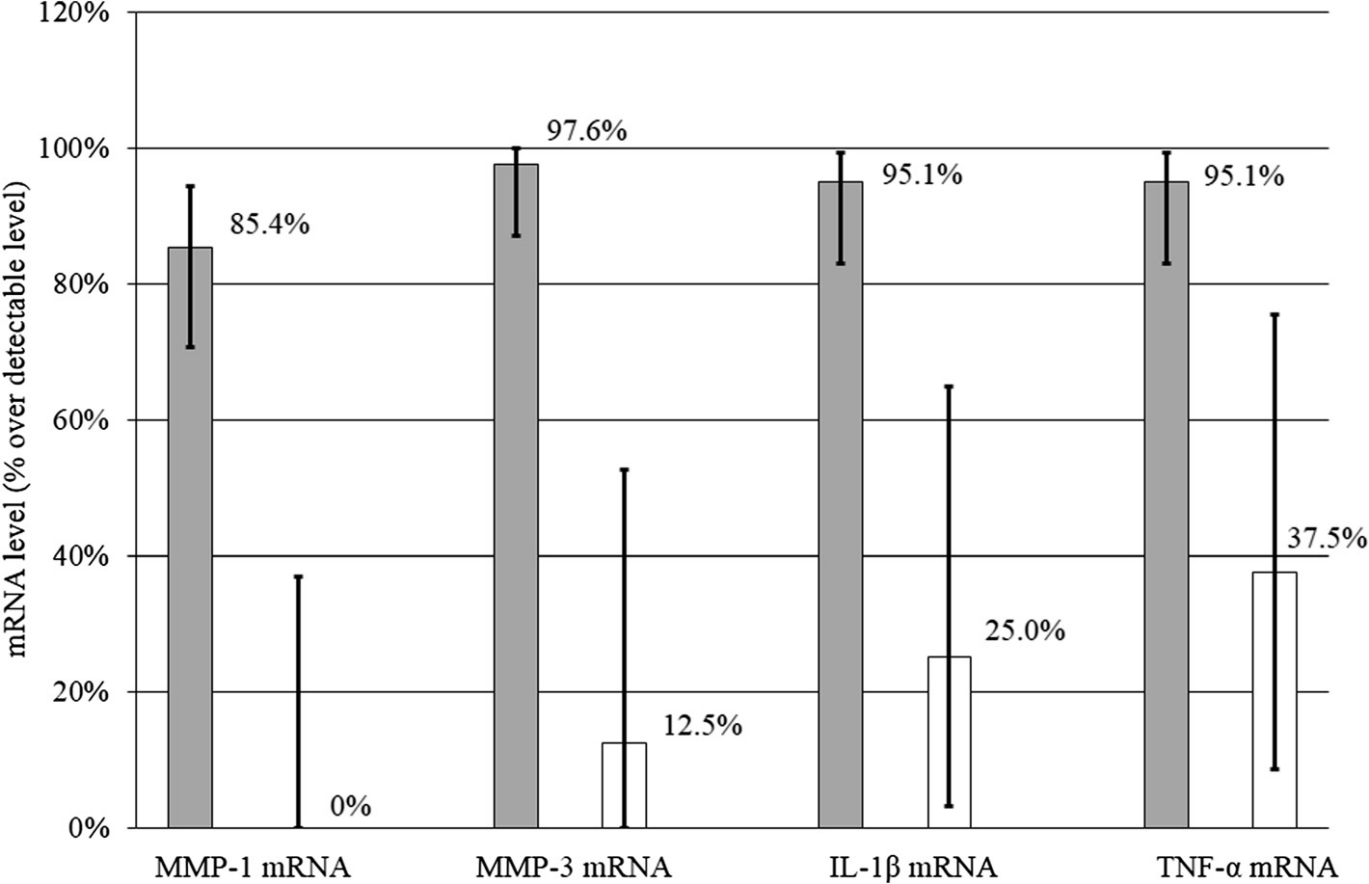
**Frequency of detectable expressions of MMP-1, MMP-3, IL-1β and TNF-α within synovial fluid (SF) yield from affected patients (OA) and controls.** Corresponding bar charts are provided with 95% confidence intervals.

Frequency of detectable expression was 85.4% (OA) versus 0% (control) for MMP-1, 97.6% (OA) versus 12.5% (control) for MMP-3, 95.1% (OA) versus 25.0% (control) for IL-1β and 95.1% (OA) versus 37.5% (control) for TNF-α; the corresponding bar charts are given in Figure 1.

Furthermore, a strong correlation appeared between expression levels of MMP-1 and MMP-3 among OA patients (r = 0.856) while no such correlation was found between age, gender or BMI and the expression levels investigated.

## Discussion

Inflammation plays a pivotal role in the pathogenesis of OA [4]. However, the cellular origin of inflammatory mediators and catabolic enzymes remains an area of speculation [14]. The aim of this study was to quantify and compare inflammatory and degradative markers on mRNA levels in cells floating in the SF of OA patients and healthy controls. The selection of TNF-α, IL-1β, MMP-1 and MMP-3 represents well-established inflammatory mediators and catabolic proteinases in OA [15,18]. The present study demonstrates that there is significantly more IL-1β, MMP-1 and MMP-3 mRNA transcribed in SF cells of OA patients compared to healthy controls. The mean age of OA patients was slightly older compared to the donor group and older age could lead to a different onset of inflammatory response. Furthermore, there was a difference in mean BMI comparing OA patients and donors. Higher BMI causes higher joint load and one could imagine that this might interfere with the inflammatory response of the investigated joints. However, the immense differences between the two groups concerning frequencies of detectable expression of IL-1β, MMP-1 and MMP-3 are not likely to be caused by the slight differences of patient age or BMI.

Increased levels of active IL-1β have already been found in SF of OA patients using ELISA [21-24]. Similar findings were made analyzing OA cartilage and synovial tissue using immunohistochemistry [25-27]. To our knowledge, this is the first study to analyze mRNA levels of IL-1β in SF cells of OA patients. Our data demonstrate a high inflammatory activity of SF cells via IL-1β. However, it is not clear to what extent these cells act synergistically with cells within the synovial tissue and cartilage. Furthermore, IL-1β’s mRNA still has to be translated and IL-1β’s inflammatory potential is influenced by the presence of IL-1β-converting enzyme, allocation of suitable receptors and other inflammatory processes. Our analysis of TNF-α mRNA concentration showed a wide range of results within the group of healthy controls and no significant difference to our OA group (*P* = 0.077). Although TNF-α is accepted as one of the two most important inflammatory cytokines in OA today, conflicting reports are found in the literature. Early studies showed highly inconsistent levels of TNF-α expression in SF and synovial tissue of OA patients [24,28-30]. Furthermore, TNF-α levels in SF significantly correlate with clinical symptoms but not with radiographic findings of OA [31]. An answer to these contrary reports might be variations in number and susceptibility of chondrocyte TNF-α receptors, the presence of soluble TNF-α receptors and activity of TNF-α-converting enzymes [24]. Alternatively, TNF-α-expressing cells such as macrophages could mainly be localized in the synovial tissue [32].

IL-1β and TNF-α stimulate the production of cartilage-degrading enzymes [33,34]. As such, MMPs are of particular interest because they are considered as the major degradative enzymes in OA [11]. Augmented MMP levels were found in SF of OA patients and they are expressed by lining cells, neutrophils, macrophages and chondrocytes in the synovial tissue [35-38]. Moreover, our data indicate that a significant amount of MMPs is present within the synovial fluid. Again, it is not clear whether these MMPs are produced by cells in the surrounding tissues or by cells within SF. First and foremost, our data indicate that cells in the SF are significant contributors. Thus, this study gives valuable information about potential treatment options including replacement of SF. Arthroscopic joint irrigation has been reported to cause significant release of pain in early studies. Livesely and colleagues showed more pain relief after arthroscopic lavage and physiotherapy compared to physiotherapy alone [39]. In contrast, Moseley and co-workers showed no difference between arthroscopic irrigation and sham operations of OA knee joints, nor regarding pain or function [40]. However, patients in the latter study were predominantly male and younger than our cohort leading to the thought that only certain patients may profit from arthroscopic irrigation. Removing inflamed SF at an early OA stage might inhibit the start of the self-escalating inflammatory cycle whereas joint lavage of late-stage OA might have no or only mild/temporary effects.

Early diagnosis of OA presents another clinical challenge and is crucial for disease prevention. Conventional diagnostic tools such as radiographic and magnetic resonance imaging (MRI) showed limited sensitivity or are time-consuming and expensive [41]. Analysis of cartilage matrix molecules in body fluids is still only used as research tool and gene expression profiles in peripheral blood leukocytes showed ambivalent cytokine levels [4,42]. Alam and colleagues successfully showed in a canine animal model that biomarkers in SF such as tartrate-resistant acid phosphatase, MMP-2, and tissue inhibitor of MMP-2 can be used for diagnosis of early OA [43]. Another canine animal model by Garner and co-workers showed high sensitivity and specificity of IL-8 and monocyte chemoattractant protein-1 in SF to surgically-induced OA. Our data show that mRNA of IL-1β, MMP-1 and -3 in SF is a sensitive indicator for symptomatic OA [44]. Further research should evaluate whether these markers are also able to detect early or non-symptomatic OA and whether they can function as a prognostic tool.

## Conclusion

Our findings show that SF of OA patients contains cells that actively promote inflammation via expression of proinflammatory mediators and joint-degrading enzymes. Although origin and type of cells still have to be investigated, this may offer an explanation as to why removal of OA SF can cause OA symptom relief. Furthermore, qPCR-based analysis of SF might offer a promising diagnostic tool and the data presented here lead to speculation towards potential pharmacologic OA intervention on a biological basis.

## Abbreviations

OA: osteoarthritis
mRNA: messenger ribonucleic acid
SF: synovial fluid
TNF: tumor necrosis factor
IL: interleucin
MMP: matrix metalloproteinase
qPCR: quantitative polymerase chain reaction
BMI: body mass index
JNK: c-Jun N-terminal kinase
MAPK: Mitogen-activated protein kinase
NF-κB: nuclear factor 'kappa-light-chain-enhancer' of activated B-cells
MCP: monocyte chemotactic protein
CCL: chemokine ligand
dNTP: desoxy ribonukleotide triphosphate
RT: reverse transcriptase
cDNA: complementary desoxyribonucleic acid
BLAST: Basic Local Alignment Search Tool
GAPDH: glyceraldehyde 3-phosphate dehydrogenase
ELISA: enzyme linked immunosorbent assay
MRI: magnetic resonance imaging
